# Li(Cd,Mn)P: a new cadmium based diluted ferromagnetic semiconductor with independent spin & charge doping

**DOI:** 10.1038/s41598-019-43754-x

**Published:** 2019-05-16

**Authors:** W. Han, B. J. Chen, B. Gu, G. Q. Zhao, S. Yu, X. C. Wang, Q. Q. Liu, Z. Deng, W. M. Li, J. F. Zhao, L. P. Cao, Y. Peng, X. Shen, X. H. Zhu, R. C. Yu, S. Maekawa, Y. J. Uemura, C. Q. Jin

**Affiliations:** 1 Beijing National Laboratory for Condensed Matter Physics, and Institute of Physics, Chinese Academy of Sciences, Beijing, 100190 China; 2 School of Physics, University of Chinese Academy of Sciences, Beijing, 100190 China; 3 Department of Physics and Electronic Engineering, Hebei Normal University for Nationalities, Chengde, 067000 China; 4 Kavli Institute for Theoretical Sciences & CAS Center for Excellence in Topological Quantum Computation, University of Chinese Academy of Sciences, Beijing, 100190 China; 5 Advanced Science Research Center, Japan Atomic Energy Agency, Tokai, 319-1195 Japan; 6 Department of Materials Science & Engineering, Sichuan University, Chengdu, China; 7 Department of Physics, Columbia University, New York, New York 10027 USA; 8 Songshan Lake Materials Laboratory, Dongguan, Guangdong 523808 China

**Keywords:** Ferromagnetism, Semiconductors

## Abstract

We report a new diluted ferromagnetic semiconductor Li_1+y_(Cd,Mn)P, wherein carrier is doped via excess Li while spin is doped by isovalence substitution of Mn^2+^ into Cd^2+^. The extended Cd 4*d*-orbitals lead to more itinerant characters of Li_1+y_(Cd,Mn)P than that of analogous Li_1+y_(Zn,Mn)P. A higher Curie temperature of 45 K than that for Li_1+y_(Zn,Mn)P is obtained in Li_1+y_(Cd,Mn)P polycrystalline samples by Arrott plot technique. The p-type carriers are determined by Hall effect measurements. The first principle calculations and X-ray diffraction measurements indicate that occupation of excess Li is at Cd sites rather than the interstitial site. Consequently holes are doped by excess Li substitution. More interestingly Li_1+y_(Cd,Mn)P shows a very low coercive field (<100 Oe) and giant negative magnetoresistance (~80%) in ferromagnetic state that will benefit potential spintronics applications.

## Introduction

Diluted ferromagnetic semiconductors have received extensive attention because of their potential applications for spintronics devices^1–3^. For the prototypical III-V diluted ferromagnetic semiconductors, such as (Ga,Mn)As, substitution of divalent Mn^2+^ into trivalent Ga^3+^ results in coupled spin & charge doping, which makes individual control of spin and charge difficult^4^. Additionally, the heterovalent substitution of Mn^2+^ into Ga^3+^ also leads to severely limited chemical solubility, and results in the specimens only available as thin films and sensitive to preparation methods and annealing treatments^5,6^. The coupled spin and charge is an obstacle not only for fundamental understanding of ferromagnetic mechanism but also for effective improvement of controllable Curie temperature (*T*_*C*_).

Recently a series of new generation diluted ferromagnetic semiconductors, *e*.*g*. “111” type Li(Zn,Mn)As and Li(Zn,Mn)P, “122” type (Ba,K)(Zn,Mn)_2_As_2_ and “1111” type (La,Ca)(Zn,Mn)SbO, have been discovered to overcome the aforementioned difficulties^7–16^. In these new types of diluted ferromagnetic semiconductors spin is doped via isovalent substitution of (Zn^2+^,Mn^2+^), while charge is provided by off-stoichiometry of Li in the “111” type diluted ferromagnetic semiconductors or by heterovalent substitution of cations in the “122” and “1111” diluted ferromagnetic semiconductors. With the advantage of independent spin and charge doping, a record of controllable *T*_*C*_ of 230 K is achieved in (Ba,K)(Zn,Mn)_2_As_2_^10,17^. Furthermore, a number of progresses of these new generation diluted ferromagnetic semiconductors have been made on both fundamental studies and potential applications^18–22^. Large size single crystals and single-phase thin films of (Ba,K)(Zn,Mn)_2_As_2_ have been grown^23–25^. Based on single crystal samples, a Mn-impurity band is identified by angle-resolved resonance photoemission spectroscopy, demonstrating strong hybridization between Mn 3*d*- and As 4*p*-orbitals^26^. The magnetic pair distribution function measurements discover presence of robust nearest-neighbor ferromagnetic alignment of Mn spins along the *c* axis even well above *T*_*C*_^27^. Andreev reflection junction is fabricated with single crystal (Ba,K)(Zn,Mn)_2_As_2_. The obtained spin polarization rate of 66% is comparable to the prototypical diluted ferromagnetic semiconductors, suggesting large potentials of application in these new generation diluted ferromagnetic semiconductors^28–30^.

Improvement of *T*_*C*_ is always a fundamental issue for diluted ferromagnetic semiconductor materials. As well known, *T*_*C*_ depends on the *p-d* exchange between carriers and Mn ions. Generally, greater *p-d* exchange can be reached by shortening bond length in Mn-ligand. However among the “111” diluted ferromagnetic semiconductor materials, Li(Zn,Mn)P has lower hole concentration and lower *T*_*C*_ than those of Li(Zn,Mn)As. Generally, ferromagnetism in a diluted magnetic semiconductor is mediated by itinerated carriers. Thus a higher *T*_*C*_ is expected with further carrier doping in Li(Zn,Mn)P. However, further improvement of carrier concentration by changing the Li concentration is invalid in Li(Zn,Mn)P. To overcome the difficulty we make a new diluted ferromagnetic semiconductor compound Li(Cd,Mn)P, where the extended Cd 4*d*-orbitals lead to more itinerant characters of Li(Cd,Mn)P than that of analogous Li(Zn,Mn)P. Li(Cd,Mn)P is expected to have larger hole concentration and consequently higher *T*_*C*_ than Li(Zn,Mn)P. In this article we report the synthesis and characterizations of the Cd-based diluted ferromagnetic semiconductor, Li(Cd,Mn)P.

## Results and Discussion

### Crystal structure

**Figure 1:**
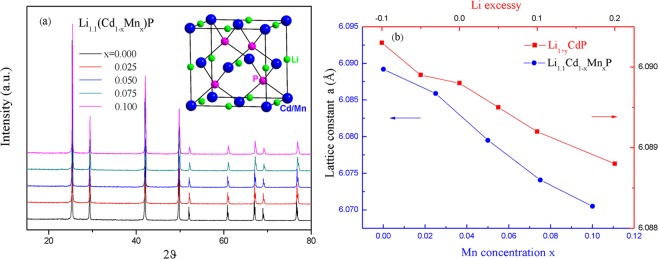
The structure characterizations. (**a**) Powder XRD patterns of Li_1.1_(Cd_1−x_Mn_x_)P with various Mn, inset: crystal structures of cubic LiCdP. (**b**) Lattice constant of Li_1.1_(Cd_1−x_Mn_x_)P (blue symbols, bottom horizontal axis) and Li_1+y_CdP (red symbols, top horizontal axis).

Figure 1(a) shows powder X-ray diffraction (XRD) patterns for the samples Li_1.1_(Cd_1−x_Mn_x_)P (x = 0.025, 0.05, 0.075 and 0.1). Parent phase LiCdP and doped samples Li(CdMn)P crystallize into a zinc-blende like structure with the space group of *F*-43*m*, as shown in the inset of Fig. 1(a). The lattice parameter *a* = 6.089(2) Å for parent phase LiCdP is consistent with the previous report^31^ and larger than LiZnAs (*a* = 5.940(2) Å) and LiZnP (*a* = 5.756(1) Å).

Homogeneity and real atomic ratios are studied with energy dispersive analysis (EDX). Except the light element Li, the atomic ratios of Cd, Mn and P are measured. The results show that all the detected elements are well distributed and their ratios are consistent with the nominal compositions (see Supplementary Fig. S1 and Tables S1 and S2). Thus the nominal compositions are used in this article for simplification. The high resolution transmission electron microscope (TEM) measurements were performed on a typical sample, the heaviest doped sample Li_1.1_Cd_0.9_Mn_0.1_P. The results don’t show any trace of defect or cluster (Fig. S3). The changes of lattice parameters with varying Li and Mn concentration in Li_1+y_(Cd_1−x_Mn_x_)P are shown in Fig. 1(b). The monotonic changes of lattice parameter with increasing Li and Mn suggest successful chemical doping. The lattice shrinks with Mn substitution due to smaller radius of Mn^2+^ (0.66 Å) than that of Cd^2+^ (0.78 Å)^32^. The decrease of lattice parameter with increasing Li concentration will be discussed later.

### Magnetic properties

**Figure 2:**
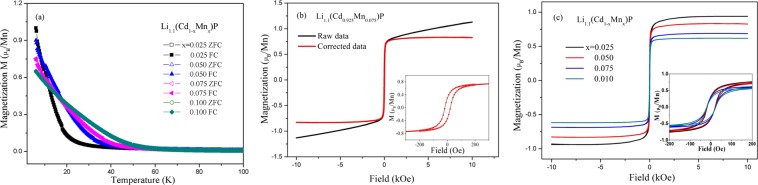
Magnetism of Li_1.1_(Cd_1−x_Mn_x_)P with x = 0.025–0.10. (**a**) M(T) under applied field H = 2 kOe with ZFC and FC procedures. (**b**) M(H) of Li_1.1_(Cd_0.925_Mn_0.075_)P at T = 6 K, measured under applied field H up to 1 T, showing hysteresis loop before and after subtraction of linear field-dependent component. Inset: hysteresis in small field regions. (**c**) M(H) curves after subtraction of the field-dependent component of Li_1.1_(Cd_1−x_Mn_x_)P with x = 0.025–0.10. Inset: corresponding M(H) curves in small field regions.

Parent phase LiCdP is diamagnetic. Single excess Li- and Mn-doping just makes the compound Pauli paramagnetic and paramagnetic (see Supplementary Fig. S2). Ferromagnetism emerges only in Li and Mn co-doped compounds in which excess Li and (Zn,Mn) substitution provide carrier and spin, respectively. The temperature dependence of magnetization (*M*(*T*))for Li_1.1_(Cd_1−x_Mn_x_)P present upturns on lowering temperature, clear signatures of ferromagnetic transition, as shown in Fig. 2(a). Note that no visible difference is detected between field cooling (FC) and zero field cooling (ZFC) modes. Among all possible impurities, only Mn_3_O_4_ has a comparable *T*_*C*_ = 42 K. In contrary, *T*_*C*_ of Li(Cd,Mn)P compounds changes from 15 K to 45 K according to their composition, ruling out the possibility of Mn_3_O_4_. The hysteresis curve (*M*(*H*))of Li_1.1_(Cd_0.925_Mn_0.075_)P at *T* = 6 K is plotted in Fig. 2(b) as a typical example. It exhibits ferromagnetic behavior with a small linear field-dependent component, which should be due to remaining paramagnetic spins^9^. Because Mn cations are randomly distributed in material, some Mn cations which locate far away from each other cannot be mediated by carries to form long range ferromagnetic order and consequently lead to local paramagnetic spins. The inset of Fig. 2(b) shows a small coercive field (*H*_*C*_ < 100 Oe) that is promising for spin manipulation. After subtracting the linear field-dependent component, the magnetic hysteresis loops of Li_1.1_(Cd_1−x_Mn_x_)P (x = 0.025, 0.05, 0.075 and 0.10) specimens at 6 K are shown in Fig. 2(c). Because the magnetizations doesn’t saturate even up to 1 T, after subtraction the magnetizations at 6 K and 1 T (*M*_6K,1T_) is used to approximately represent saturation magnetizations in following discussions. The *M*_6K,1T_ = 0.94, 0.83, 0.69, 0.62 μ_B_/Mn for samples of x = 0.025, 0.05, 0.075 and 0.10 respectively. They are smaller than that of (Ga,Mn)As^4^ and Li(Zn,Mn)As^7^ possibly due to the competition between the long range ferromagnetic ordering and the short range antiferromagtic interactions. The tendency that the M_6K,1T_ reduces with increasing Mn doping levels has also been found in many magnetic ions doped materials^33^. One rational reason is the competition between ferromagnetic interaction of Mn mediated by carriers and antiferromagnetic coupling of Mn pairs in the nearest neighbor sites, as discussed in “111” and “122” diluted ferromagnetic semiconductors^7–9,12^.

**Figure 3:**
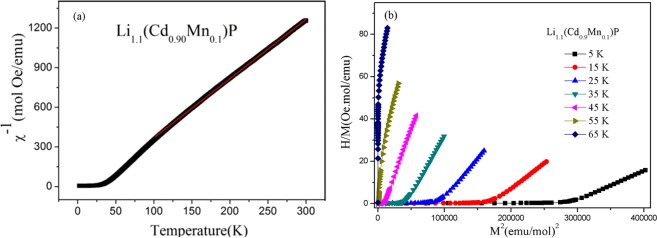
The ferromagnetic ordered temperature of the diluted magnetic semiconductor. (**a**) Inverse susceptibility dependence of temperature for Li_1.1_(Cd_0.9_Mn_0.1_)P (black line) and Curie-Weiss fit (red line). (**b**) Arrott plots at various temperatures above and below Tc for Li_1.1_(Cd_0.9_Mn_0.1_)P, shows the ferromagnetic transition at Tc = 45 K.

In the paramagnetic region, the susceptibility can be well fitted by the Curie-Weiss law, as shown in Fig. 3(a). The obtained Weiss temperature is 17.8(2) K, demonstrating the ferromagnetic interaction between Mn. To precisely determine the *T*_*C*_, Arrott plot method is performed. In Fig. 3(b), *H*/*M* versus *M*^2^ is plotted over the temperature range of 5–65 K. The isotherm at the Curie point is supposed to be a straight line passing through the origin. In this way, *T*_*C*_ is determined as 45 K for Li_1.1_(Cd_0.9_Mn_0.1_)P. The *T*_*C*_ for the other samples are obtained in similar way. Table 1 lists *T*_*C*_ and *M*_6K,1T_ for various compositions of Li_1+y_(Cd_1−x_Mn_x_)P. When Mn concentration is fixed, one can note that excess Li initially improves both *T*_*C*_ and *M*_6K,1T_ within low Li concentration but then suppresses ferromagnetic order when y = 0.2. On the other hand, except samples with overdoped Li (y = 0.2), increasing Mn enhances the *T*_*C*_ but declines the average local moments on Mn (*M*_6K,1T_).

**Table 1:** The Curie temperature (*T*_*C*_) and M_6K,1T_ to approximately represent saturation magnetizations of Li_1+y_(Cd_1−x_Mn_x_)P.

*T*_*C*_ (K)/M_6K,1T_ (µ_B_/Mn)	y = 0.05	y = 0.1	y = 0.2
x = 0.025	23/0.84	21/0.94	15/0.71
x = 0.05	27/0.72	27/0.83	16/0.37
x = 0.075	31/0.60	35/0.69	16/0.18
x = 0.10	36/0.56	45/0.62	15/0.26

### Electrical transport properties

**Figure 4:**
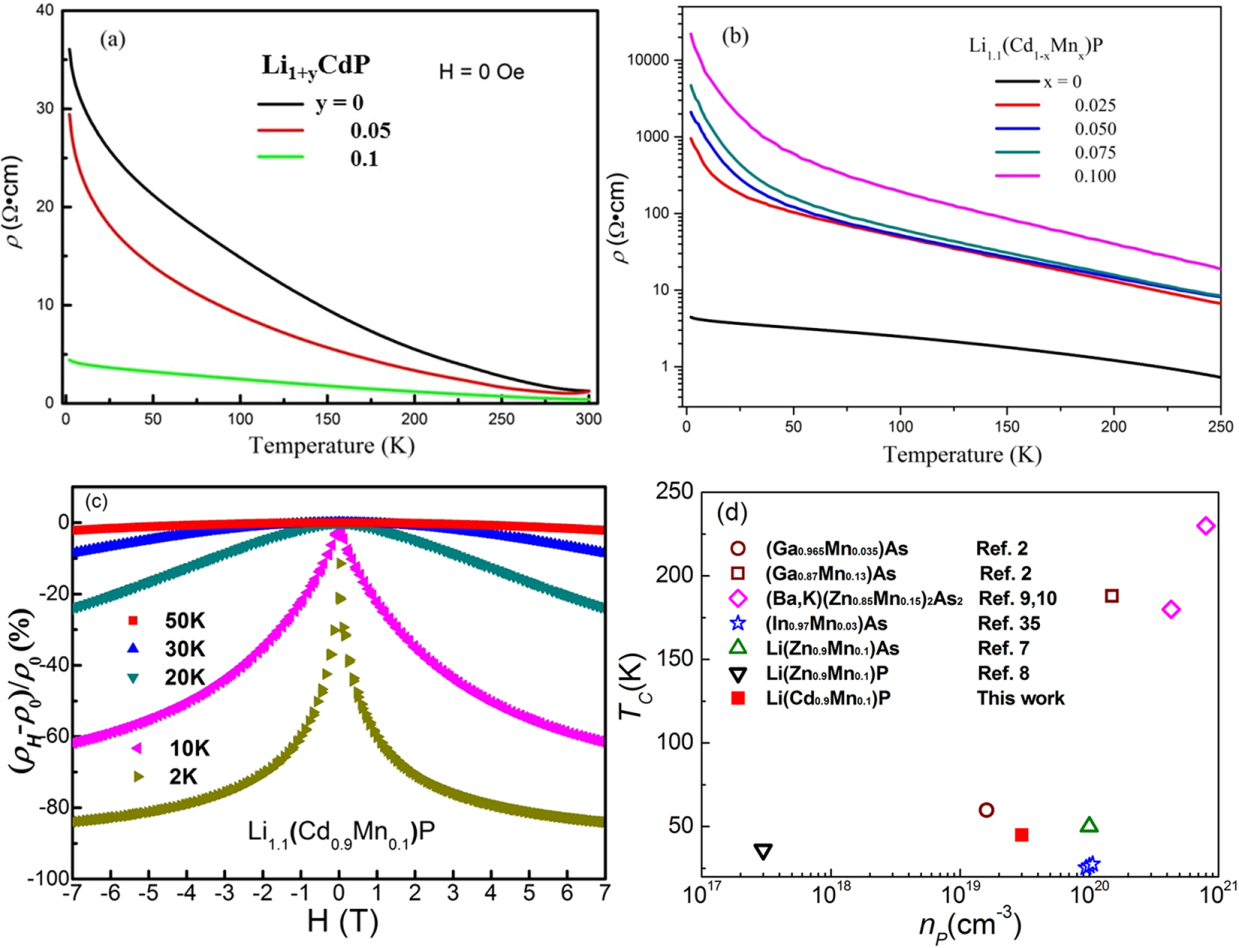
Transport properties of Li_1+y_(Cd_1−x_Mn_x_)P: (**a**) ρ(T) of Li_1+y_CdP with y = 0, 0.05 and 0.1. (**b**) ρ(T) of Li_1.1_(Cd_1−x_Mn_x_)P with x = 0, 0.025, 0.05, 0.075 and 0.1. (**c**) Magnetoresistance of Li_1.1_(Cd_0.9_Mn_0.1_)P at different temperatures. (**d**) Correlation between *T*_*C*_ and the hole concentration for several “111”, “122” new types of diluted ferromagnetic semiconductors and typical III-V diluted ferromagnetic semiconductors.

Figure 4(a) shows the temperature-dependent resistivity (*ρ*(*T*)) for a series of Li_1+y_CdP (y = 0, 0.05 and 0.1). Consistent with ref.^31^, the parent compound LiCdP present semiconducting conduction^31^. Excess Li doping decrease resistivity, suggesting the effective doping of carriers. Figure 4(b) shows that at the entire temperature range the resistivity of Li_1.1_(Cd_1−x_Mn_x_)P increases with increasing Mn concentration, caused by localization effect. Although carriers introduced by excess Li doping are originally itinerant, they will be weakly bound to the Mn local spin moments and then partially lose their mobility^34^. The similar conducting behavior has been observed in (Ba,K)(Zn,Mn)_2_As_2_. The localization of the carriers has been confirmed by Mn *K β* x-ray emission spectroscopy measurements which indicates that Mn local spin moments traps holes and in turn is declined by hole doping^20^.

A giant negative magnetoresistance (MR) is observed in all of the ferromagnetic Li(CdMn)P samples. Figure 4(c) shows the MR of Li_1.1_(Cd_0.9_Mn_0.1_)P specimen as a typical example. The value of negative MR (about −80%) is at least twice larger than those of analogues Li(Zn,Mn)As and Li(Zn,Mn)P^7,8^. The negative MR may result from reduction of spin-dependent scattering by aligning the spins under applied field. The Hall effect measurements of all the samples show p-type carriers. Hole concentration (*n*_*p*_) of the parent phase is ~10^17^ cm^−3^ at 200 K. More holes are doped by excess Li substitution in Li_1.1_CdP which has *n*_*p*_ = 3.8 × 10^19^ cm^−3^ at 200 K. In ferromagnetic sample Li_1.1_(Cd_0.95_Mn_0.05_)P, 5% Mn doping slightly decreases to *n*_*p*_ = 2.7 × 10^19^ cm^−3^ at 200 K. At lower temperature the resistivity was too large, any small misalignment of the two Hall contacts picks up a longitudinal resistivity signal and this leads to great difficulty in the Hall effect measurement (see Supplementary Fig. S4). The relationships between hole concentration and Curie temperature of “111” diluted ferromagnetic semiconductors and other diluted ferromagnetic semiconductor systems are plotted in Fig. 4(d)^2,7–10,35^. From Li(Zn,Mn)P to Li(Cd,Mn)P, the hole concentration is considerably increased, and consequently the *T*_*C*_ is improved from 34 K to 45 K. As the Zener model predicted, the ferromagnetism is mediated by carriers, and the Curie temperature is positive correlated with hole concentration.

### Theoretical analysis

In order to have insight into the origin of hole carrier, we performed calculation on electronic structures with density functional theory (DFT). Calculation shows that the band structure of LiCdP is very similar to that of LiZnP. The band structure of parent phase LiCdP is shown in Fig. S5. The obtained direct energy gap is 0.59 eV. With the quantum Monte Carlo (QMC) simulation of the Anderson impurity model, the impurity band level of Mn is determined as −0.35 eV.

Two possible different sites for excess Li are discussed in the calculations, (i) the interstitial site Li_I_ and (ii) the Cd-substitutional site Li_Cd_. The former can provide n-type carrier and the latter will serve as a hole donor. We calculate the formation energy for the two excess Li-sites, respectively. Since Mn at Cd-substitutional site Mn_Cd_ does not introduce any carriers, we study the excess Li in Li_1+y_CdP for simplification. According to previous work^36^, the formation energy is given by E_formation_ = E_T_ − n_Li_μ_Li_ − n_Cd_μ_Cd_ − n_P_μ_P_, where E_T_ is the total energy of the supercell, n_x_ is the number of x atoms in the supercell, and μ_x_ is the atomic chemical potential. It has μ_Li_ + μ_Cd_ + μ_P_ = μ_LiCdP(bulk)_. Table 2 shows formation energy for two extreme conditions, *i*.*e*., the Li-rich plus Cd-rich limit (μ_Li_ = μ_Li(bulk)_, μ_Cd_ = μ_Cd(bulk)_) and the Li-rich plus P-rich limit (μ_Li_ = μ_Li(bulk)_, μ_P_ = μ_P(bulk,black)_). Under both conditions, compounds with Li_Cd_ have lower formation energy. The experimental condition must be between these two extreme conditions. It recalls the reduction of lattice parameter with excess Li doping found in XRD measurements. The excess Li at interstitial site is supposed to stretch the lattice. In contrary, substitution of Li into Cd should shrink the lattice due to the smaller Li^+^ radius (0.59 Å) than Cd^2+^ (0.78 Å)^32^. Thus we argue that in Li(Cd,Mn)P the excess Li atoms prefer to occupy Cd-substitutional sites Li_Cd_, and thus create the p-type carriers.

**Table 2:** Formation energy for excess Li atom at different sites, obtained by DFT calculations^36^.

LiCdP with excess Li	Formation energy (Li-rich and Cd-rich limit)	Formation energy (Li-rich and P-rich limit)
Interstitial Li (supercell Li_28_Cd_27_P_27_)	2.13 eV	2.13 eV
Li at Cd site and Cd is removed (supercell Li_28_Cd_26_P_27_)	0.22 eV	−1.14 eV

## Conclusions

In this work, “111” type Cd-based Li(Cd,Mn)P has been designed to achieve high Curie temperature. In Li(Cd,Mn)P replacement of Zn by Cd successfully compensates for the high hole binding energy in the Li(Zn,Mn)P by increasing Mn-P bond length. As a result, Li(Cd,Mn)P has enlarged the hole concentration, amplified the effective *p-d* exchange and more importantly a Curie temperature of 45 K which is one third higher than *T*_*C*_ of Li(Zn,Mn)P. In addition, the observed properties in Li(Cd,Mn)P, such as the low coercive field and the giant negative magnetoresistance, are favorable for future applications. In short, the successful prediction and fabrication of Li(Cd,Mn)P open an new boulevard to tailor the ferromagnetism in diluted magnetic semiconductors.

## Method

Polycrystalline specimens of Li(Cd,Mn)P were prepared by solid state reaction with high purity elements. The stoichiometric ratios of starting materials were well mixed and pressed into pellets. All the processes were performed under the protection of high-purity Argon due to the air sensitivity of precursors and products. The pellets were sealed in Ta tubes under 0.5 bar of Argon before being sealed into evacuated quartz tubes. The samples were heated at 470 °C for 48 h. Then the products were reground and sintered at 680 °C for 48 h, followed by a quick quenching to room temperature. The recovered samples were characterized by X-ray powder diffraction (XRD) with a Philips X’pert diffractometer using Cu*Kα* radiation. Real compositions of the heavy elements (*i*.*e*. Cd, Mn and P) were determined by using energy dispersive analysis (EDX) on a commercial Scanning Electron Microscope (SEM). Microstructure was studied by high resolution Transmission Electron Microscope (TEM). The *dc* magnetic properties were examined by using Superconductivity Quantum Interference Device (SQUID, Quantum design), and transport properties were examined by Physical Property Measurement System (PPMS, Quantum design). We calculated the electronic structures by using the density functional theory (DFT) implemented in the code QUANTUM ESPRESSO^36^. The exchange-correlation interactions are described by the Perdew-Burke-Ernzerhof generalized gradients approximation (GGA), and the electronion interactions are represented by the Vanderbilt ultrasoft pseudopotentials.

## Supplementary information

The Supporting Materials
