# Supplementary material for: Li(Cd,Mn)P: a new cadmium based diluted ferromagnetic semiconductor with independent spin & charge doping
Source: Sci Rep. 2019 May 16;9:7490. doi: 10.1038/s41598-019-43754-x (PMC6522530; doi:10.1038/s41598-019-43754-x)
Supplement: Supplementary file 1 — The Supporting Materials [file 41598_2019_43754_MOESM1_ESM.doc]

**Supporting Information**

**Li(Cd,Mn)P: a new cadmium based diluted ferromagnetic semiconductor** **with independent spin & charge doping**

W. Han^1,2,3^, B. J. Chen^1,2^, B. Gu^4,5^, G. Q. Zhao^1,2^, S. Yu^1,2^, X. C. Wang^1,2^, Q. Q. Liu^1,2^, Z. Deng^1,2*^, W. M. Li^1,2^, J. F. Zhao^1,2^, L. P. Cao^1,2^, Y. Peng^1,6^,, X. Shen^1^, X. H. Zhu^6^, R. C. Yu^1^, S. Maekawa^5^, Y. J. Uemura^7^, C. Q. Jin^1,2,8*^

^1^*Beijing National Laboratory for Condensed Matter Physics, and Institute of Physics, Chinese Academy of Sciences, Beijing 100190, China*

^2^*School of Physics, University of Chinese Academy of Sciences, Beijing 100190, China*

^3^*Department of Physics and Electronic Engineering, Hebei Normal University for Nationalities, Chengde 067000, China*

^4^*Kavli Institute for Theoretical Sciences & & CAS Center for Excellence in Topological Quantum Computation, University of Chinese Academy of Sciences, Beijing 100190, China*

^5^*Advanced Science Research Center, Japan Atomic Energy Agency, Tokai 319-1195, Japan*

*^6^Department of Materials Science & Engineering, Sichuan University, Chengdu, China*

^7^*Department of Physics, Columbia University, New York, New York 10027, USA*

^8^ *Songshan Lake Materials Laboratory, Dongguan, Guangdong, 523808, China*

*Correspondence and requests for materials should be addressed to Z. D. (email: dengzheng@iphy.ac.cn) or C. Q. J. (email: Jin@iphy.ac.cn)

Energy dispersive analysis of X-ray and high resolution transmission electron microscope measurements

We used energy dispersive analysis of X-ray measurements (EDX) to measure the real atom ratios of heavy elements, Cd, Mn and P. The light element Li is not precisely detectable by EDAX. But its concentration evolution can be inferred from change of lattice constant.

For each sample, we probed 3 regions to testify distribution and homogeneousness. Three spectra of Li_1.1_Cd_0.9_Mn_0.1_P are shown in Figure S1. The atom ratio is summarized in Table S1. Considering the precision of the EDX, we believe the sample is homogeneous. Table S2 summarizes the obtained real element compositions of Li_1.1_Cd_0.95_Mn_0.05_P, Li_1.1_Cd_0.9_Mn_0.1_P and Li_1.15_Cd_0.85_Mn_0.1_P, which are quit close to the nominal ones.


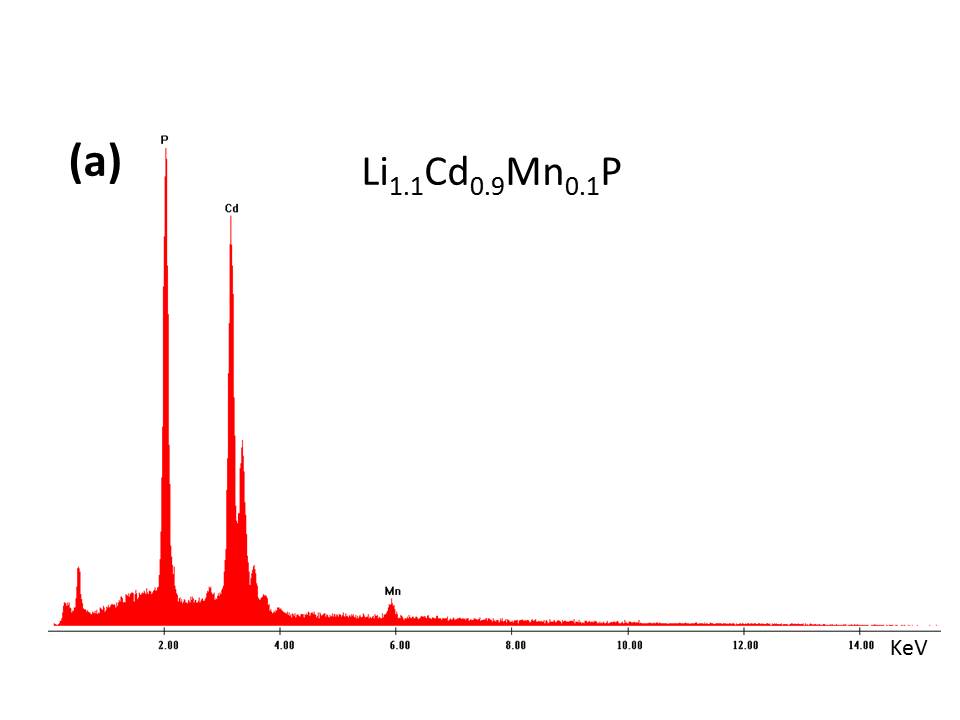


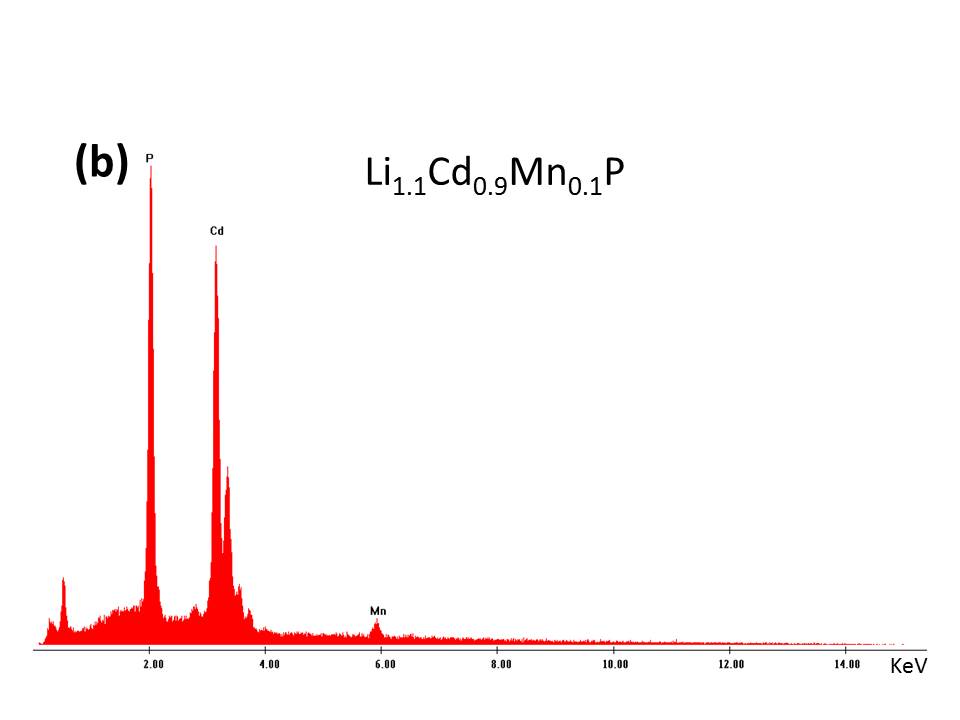


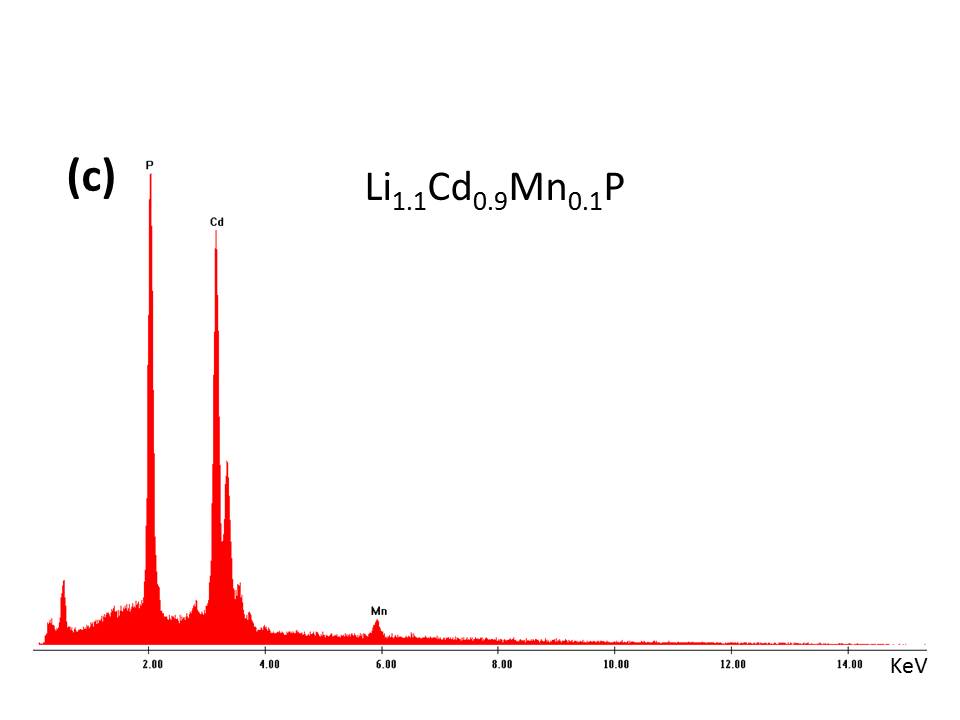


**Figure S1.** (a)-(c) The EDX spectra of the sample Li_1.1_Cd_0.9_Mn_0.1_P

**Table S1.** Summary of real composition of Li_1.1_Cd_1-x_Mn_x_P, with x = 0.05, 0.10, 0.15.

| nominal composition | measured composition by EDX |
| --- | --- |
| Li_1.1_Cd_0.95_Mn_0.05_P | Li_1.1_Cd_0.939_Mn_0.061_P |
| Li_1.1_Cd_0.9_Mn_0.1_P | Li_1.1_Cd_0.887_Mn_0.113_P |
| Li_1.1_Cd_0.85_Mn_0.15_P | Li_1.1_Cd_0.816_Mn_0.184_P |

**Table S2** Atom ratio of Cd, Mn and P of Li_1.1_Cd_0.9_Mn_0.1_P, taken from three different region s.

| element | atom ratio | | |
| --- | --- | --- | --- |
|  | region 1 | region 2 | region 3 |
| Cd | 0.882 | 0.888 | 0.891 |
| Mn | 0.118 | 0.112 | 0.109 |
| P | 1 | 1 | 1 |

We performed high resolution TEM on Li_1.1_Cd_0.9_Mn_0.1_P, the heaviest doped sample, to study the microstructure. We didn’t find defect or cluster in all grains. Two zones are shown in Figure S2(a) and S2(b) as typical examples.


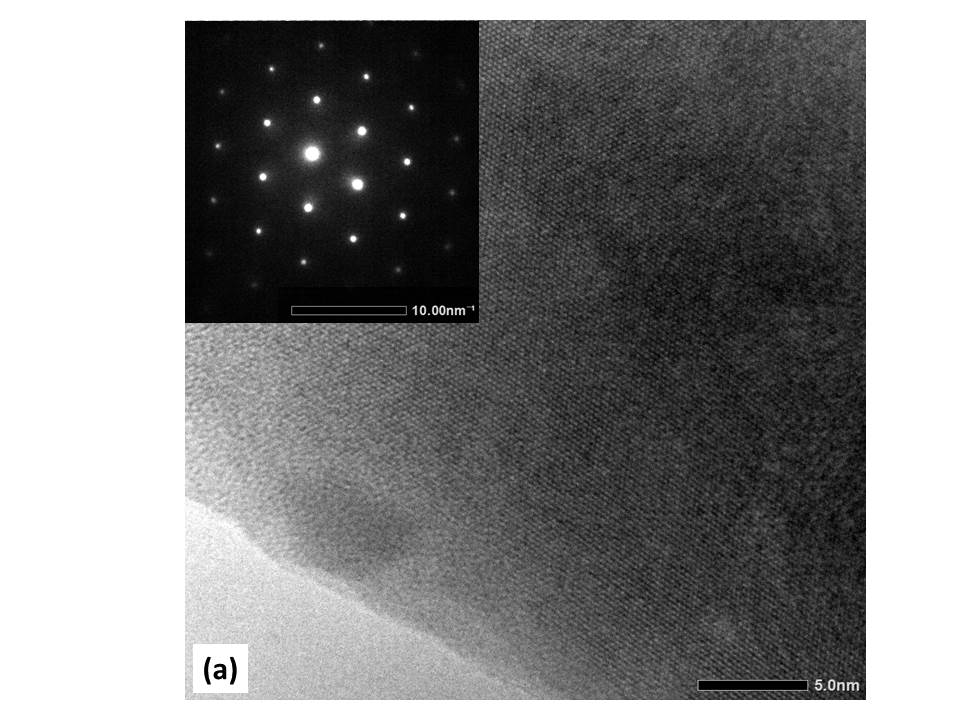


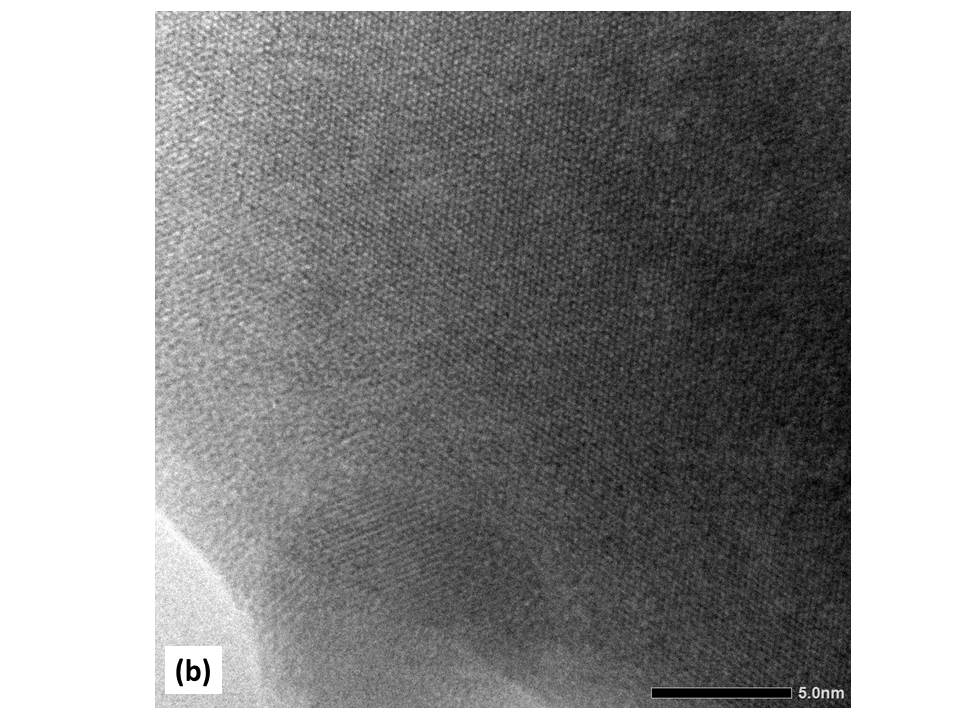


**Figure S2** (a) and (b) High resolution TEM of Li_1.1_Cd_0.9_Mn_0.1_P. Darkness is from the thickness of the grain. Inset of (a) is SAED pattern.

Magnetic measurements of LiCdP, Li_1.1_CdP and LiCd_0.9_Mn_0.1_P

To clarify the magnetic properties of parent phase, excess Li-doped samples and Mn-doped samples, temperature-dependent magnetization of LiCdP, Li_1.1_CdP and Li(Cd_0.9_Mn_0.1_)P were measured. In most of temperature range of Fig S3(a), LiCdP is diamagnetic due to absent of unpaired *d* electron. The sudden upturn below 30 K may be caused by a small amount of paramagnetic impurity. Fig S3(b) presents Pauli paramagnetic behavior owing to extra carriers induced by excess Li. Fig S3(c) shows that Li(Cd_0.9_Mn_0.1_)P is paramagnetic down to 2K.

**
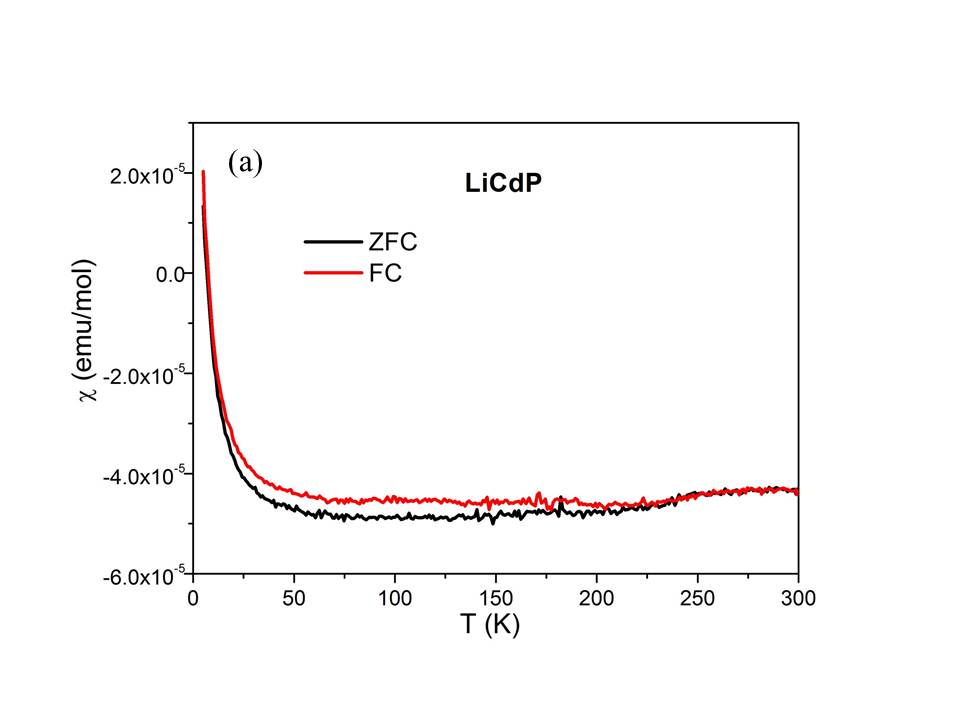
**

**
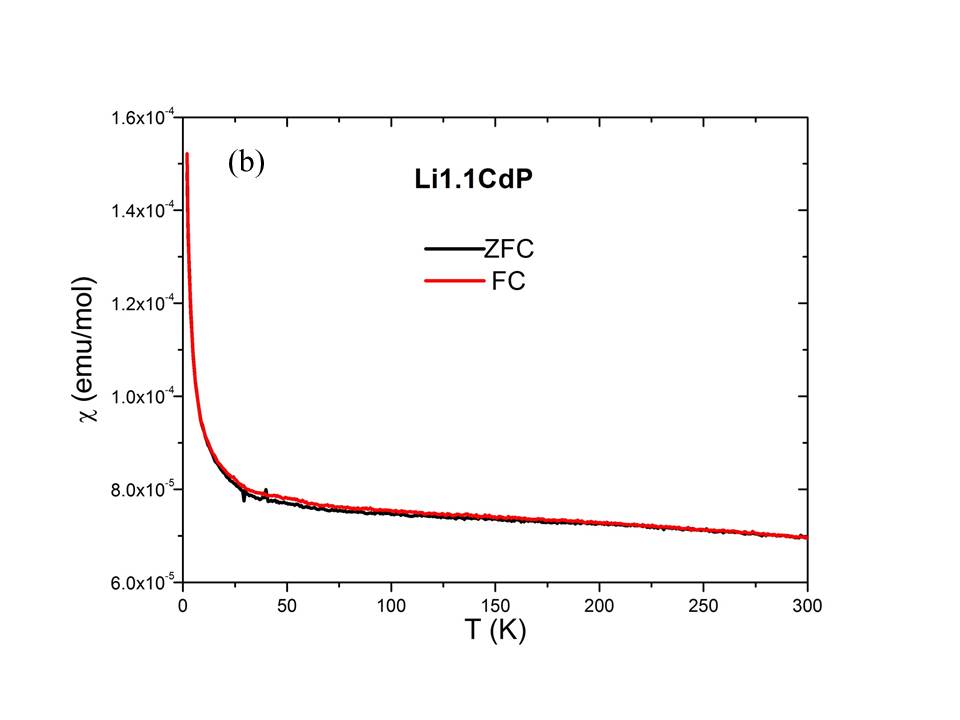
**

**
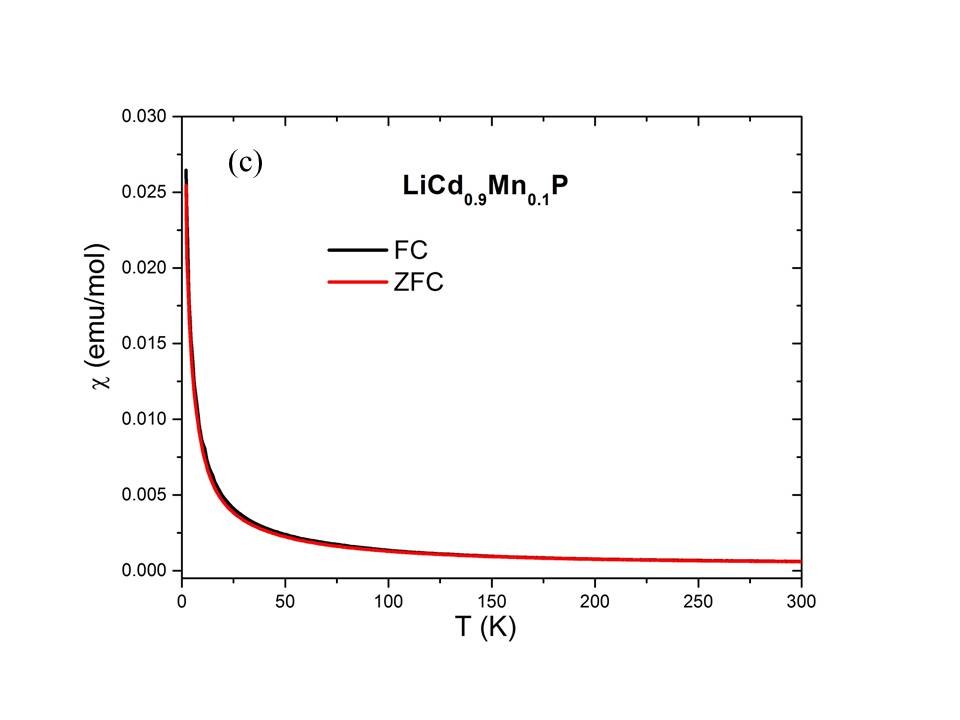
**

**Figure S3.** M(T) under applied field H = 2 kOe with ZFC and FC procedures for (a) parent phase LiCdP, (b) excess Li-doped sample Li_1.1_CdP and (c) Mn-doped sample Li(Cd_0.9_Mn_0.1_)P.

Hall Effect measurements

To determine the carrier type and concentration, Hall effect measurement is performed on a Quantum Design Physical Property Measurement System (PPMS) with standard Hall bar method.

As the resistivity value was too large at low temperature and any small misalignment of the two Hall contacts would collect a longitudinal resistivity, the Hall effect measurement became very difficult to be accomplished. Thus all of the successful measurements were performed at 200 K.

All the samples show p-type carriers. Hole concentration (*n_p_*) of the parent phase, Li_1.0_CdP, is ~10^17^cm^-3^ at 200 K. More holes are doped by excess Li substitution in Li_1.1_CdP which has *n_p_* = 3.8×10^19^cm^-3^ at 200 K (Fig. S4(a)). In ferromagnetic sample Li_1.1_(Cd_0.95_Mn_0.05_)P, 5% Mn doping slightly decreases to *n_p_* = 2.7×10^19^cm^-3^ at 200 K (Fig. S4(b)).

**Figure S4.** Hall resistivity versus magnetic field of (a) Li_1.0_CdP; (b) Li_1.1_(Cd_1-x_Mn_x_)P with x = 0, 0.05 and 0.1.

Theoretical Calculation: band structure of LiCdP

Our Density functional theory（DFT) calculation shows that the band structures of LiCdP and LiZnP are very similar except the value of band gap (Figure S5 and Table S3). With the quantum Monte Carlo (QMC) simulation of the Anderson impurity model, we have studied the Mn impurity state. In order to reproduce the Mn^2+^ (3d^5^) state in the Mn-doped LiCdP by the QMC simulation, the parameter of impurity level Ed is determined as -0.35 eV, where zero is set as the top of valencd band.


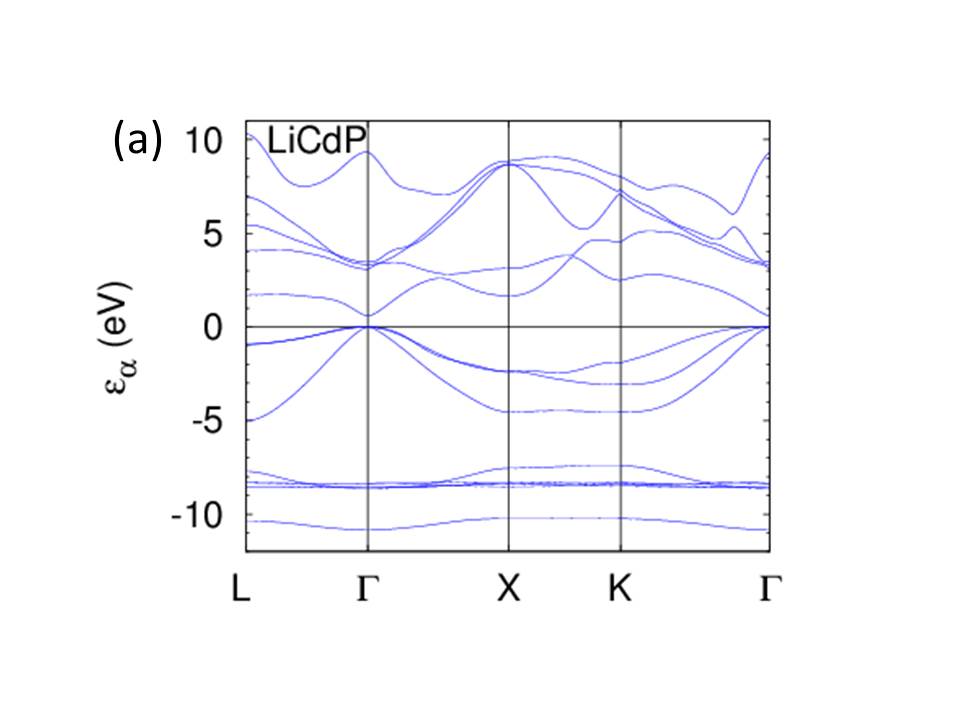


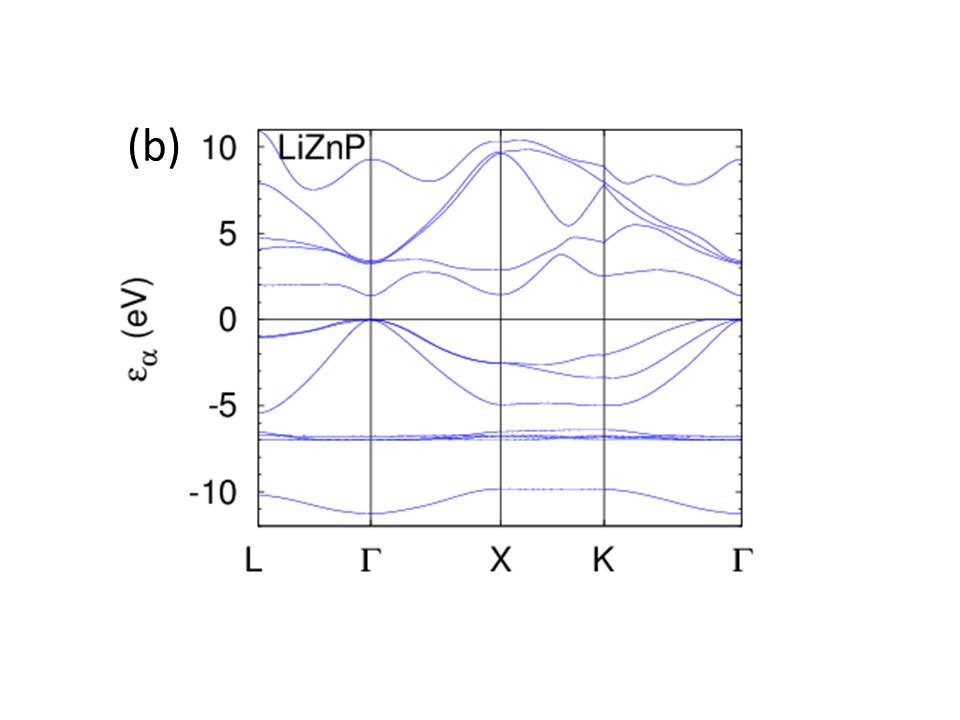


**Figure S5** Calculated band structure of LiCdP and LiZnP.

**Table S3** Calculated energy gap and impurity band level of Mn

|  | Energy Gap (eV) | | Impurity band level of Mn (eV) |
| --- | --- | --- | --- |
|  | Experiment | DFT (GGA) |  |
| LiCdP | 1.3[S1] | 0.59 | -0.35 |
| LiZnP | 2.1[S1] | 1.38 | -1.5 |

Reference

[S1] Bacewicz, R. & Ciszek, T. F. Preparation and characterization of some AIBIICV type semiconductors. *Applied Physics Letters* **52**, 1150 (1988).
